# Effects of second litter syndrome on reproductive performance in sows

**DOI:** 10.14202/vetworld.2024.1680-1684

**Published:** 2024-08-03

**Authors:** Nguyen Hoai Nam, Thepsavanh Khoudphaithoune, Do Thi Kim Lanh, Nguyen Van Thanh, Nguyen Duc Truong, Nguyen Cong Toan, Bui Van Dung, Bui Tran Anh Dao, Peerapol Sukon

**Affiliations:** 1 Department of Animal Surgery and Theriogenology, Faculty of Veterinary Medicine, Vietnam National University of Agriculture, Hanoi, Vietnam; 2 Department of Veterinary Pathology, Faculty of Veterinary Medicine, Vietnam National University of Agriculture, Hanoi, Vietnam; 3 Department of Anatomy, Faculty of Veterinary Medicine, Khon Kaen University, Khon Kaen, Thailand; 4 Research Group for Animal Health Technology, Khon Kaen University, Khon Kaen, Thailand

**Keywords:** piglets born alive, reproductive performance, second litter syndrome

## Abstract

**Background and Aims:**

The effects of second litter syndrome (SLS) on subsequent reproductive performance remain poorly understood. This study examined the impact of SLS on reproductive parameters such as piglets born alive (PBA), accumulative number of PBA (APBA), farrowing interval (FI), and risk of decreased PBA (DPBA) up to parity 5.

**Materials and Methods:**

Data on 5,464 litters were recorded from 1,507 sow cards collected on five swine farms in northern Vietnam. A linear mixed-effect model was used to analyze the effect of SLS on the PBA, APBA, and FI. A generalized linear mixed model was used to analyze the effect of DPBA in parity n on the risk of DPBA in parity n + 1.

**Results:**

About 47.8% of the sows contracted SLS (720/1507). Only APBA1-2 was significantly decreased by SLS. The APBA3-5 in SLS sows was comparable to that in non-SLS sows (41.8 vs. 41.9). Non-DPBA2 upped the risk for DPBA3 by 3.6-fold (95% confidence interval [CI]: 2.8–4.6). Moreover, non-DPBA3 increased the risk of DPBA4 (odds ratio [OR] = 2.7, 95% CI = 2.1–3.7), and non-DPBA4 increased the risk of DPBA5 (OR = 3.2, 95% CI = 2.3–4.7). The risks of developing DPBA4 and DPBA5 remained unchanged following SLS (p > 0.05). About 98.4% of sows underwent PBA fluctuations during their first five parities.

**Conclusion:**

SLS does not appear to detrimentally affect PBA, APBA, and FI in subsequent parities. Therefore, SLS sows do not necessarily have future low reproductive performance or be culled. Future investigations should explore the mechanism of alternate decrease/increase patterns in PBA.

## Introduction

Normally, the number of piglets born alive (PBA) in the second parity is greater than in the first parity [1–4]. However, many sows may have their PBA in the second parity equal to or smaller than in the first parity [5–10]. This phenomenon is called second litter syndrome (SLS) [11]. Studies conducted in America, Japan, Mexico, Brazil, Spain, and the Netherlands showed that the incidence of SLS varied from 33.3% to 56.6% [12–19]. Multiple risk factors for SLS, including a large PBA at the first parity, short lactation lengths, inadequate weight gain during initial insemination and first weaning, and short intervals between weaning and next service, have been identified [13, 16, 17, 19, 20]. The precise impact of age at first farrowing on SLS remains unclear. A study by Le Cozler *et al*. [21] supported the increased age at first farrowing to decrease the incidence of SLS in Large White × Landrace sows, whereas the result by Sanz-Fernández *et al*. [12] supported the early age of Iberian sows.

Sows with a smaller PBA in parity 2 may also have a smaller PBA in parity 3 and later parities [22], which may increase the culling risk [23, 24]. A prior investigation revealed disparate outcomes on lifetime PBA from SLS exposure [25]. Sasaki *et al*. [25] reported that sows with PBA2 decreased by 1 or increased by 3 piglets had a higher lifetime PBA than sows with PBA2 decreased by ≤2 or increased by ≥4 piglets. However, sows with PBA2 increased by ≥4 piglets had a similar lifetime PBA compared with sows that had PBA2 decreased by 2–3 piglets [25]. Such results prevent us from concluding the effect of SLS on lifetime PBA. Some studies have categorized sows as “low PBA1-medium PBA2” (LM) and “medium PBA1-low PBA2” (ML), which may be, to some extent, similar to non-SLS and SLS, respectively, and reported that LM and ML sows had similar accumulative piglets born alive (APBA) until parity 5 [22] or parity 10 [14]. Nevertheless, these 2 studies did not evaluate the effect of SLS on reproductive performance in sows at later parity. Information about the effect of SLS on the farrowing interval (FI) is also restricted. A previous study has shown that SLS sows have a smaller summed PBA1 + PBA2, fewer nonproductive days, and a shorter FI than non-SLS sows [18]. To go into detail, the summed PBA, the nonproductive days, and the FI between parity 1 and 2 in SLS sows were 19.5 piglets, 12.8 days, and 149.2 days, respectively, and these parameters were 19.8 piglets, 14.7 days, and 150.8 days, respectively [18].

Given the limited information about the impact of SLS on reproductive parameters, the present study aimed to investigate the effect of SLS on PBA, APBA, FI, and risk of decreased piglets born alive (DPBA) at different parities until parity 5.

## Materials and Methods

### Ethical approval

Since the retrospective study utilized farm data without interfering in any farm activities, it was exempt from ethical approval by the Ethics Committee.

### Study period and location

The study was conducted from September to December 2023 on five swine farms in Northern Vietnam.

### Data collection

The five studied farms had breeding capacities of about 500–700 Landrace × Yorkshire sows. Across the five farms, sows were fed similar industrialized gestation and lactation feeds; however, the amount of feed was not the same. Depending on the gestation stage, sows were fed about 1.8–3.5 kg feed. The difference in the amount of feed per sow per day among five farms varied from 0 to about 200 g. On the five farms, sows were artificially inseminated once per estrus cycle with Duroc boar semen. The temperature of the farms was controlled using fans, cooling pads, sprinklers, and infrared light. The temperature and humidity at the gestation and farrowing rooms were maintained below 28°C and 80%, respectively.

A request for data collection with clear objectives was sent to the veterinarians who worked on these farms. They were asked to take pictures of the paper sow cards of the sows that had farrowed at least two litters and to send these pictures to the principal investigator through the Zalo application. In total, 1712 images were retrieved. Pictures of low quality and/or unclear content that made it difficult to recognize the data were excluded from the study. Usually, the sow cards had the following information: sow identification, dates of insemination, expected farrowing, farrowing, weaning, total number of piglets born, number of PBA, number of stillbirths, number of mummies, number of piglets with deformities, and number of weaned piglets. Some sow cards had information about average piglet birth weight, weaning weight, date of sow birth, and date of first estrus. With the objectives to investigate the incidence of SLS and its effect on PBA, APBA, FI, and DPBA at different parities, sow cards with clear information on sow identification, dates of insemination and farrowing, and number born alive at each parity were included in the study, resulting in 1507 valid sow cards with 5464 litters. All sows were born between 2020 and 2022.

### Data definition

Sow identification, insemination dates, actual farrowing, and number of (PBA, piglet/litter) at different parities (from 1 to 5) from the sow cards were manually typed into a Microsoft Excel 2016 (Microsoft, Washington, USA) file. A sow with the number of PBA in parity 2 (PBA2) equal to or less than the number of PBA in parity 1 (PBA1) was considered to acquire SLS. The use of live-born piglets rather than total number of born piglets was based on a previous definition of SLS [25]. A sow with the number of PBA in parity 3 (PBA3) ≤ PBA2 was defined as a decreased number of PBA in parity 3 (DPBA3). A sow with a number of PBA in parity 4 (PBA4) ≤ PBA3 was defined as DPBA4. A sow with the number of PBA in parity 5 (PBA5) ≤ PBA4 was defined as DPBA5. Accumulative numbers of piglets born from parity 1 to parity n (APBA1-n) were the sums of all PBAs from parity 1 to parity n. The interval from farrowing one to farrowing n (FI1-n, day) was calculated by subtracting the date of farrowing one from the date of farrowing n.

### Statistical analysis

For continuous outcomes (PBA, APBA, and FI), linear mixed-effect models were used to compare each outcome between SLS and non-SLS sows. The formula for this model is expressed as follows: Yij = μ + SLSi + Fj + εij, where Yij = dependent variable, μ = overall mean, SLSi = fixed effect of SLS, Fj = random effect of the farm, and εij = residual error. The effect size and its 95% confidence interval (95% CI) for each outcome were also calculated. For a binary outcome, Generalized Linear Mixed Models were used to compute the odds ratio (OR) for the risk analysis of DPBA3, DPBA4, and DPBA5 in different groups of sows. The formula for this model is expressed as follows: logit (pij) = β0 + β1DPBAi + Fj + ɛij, where pi = probability of the binary outcome, β0 = intercept, β1DPBAi = fixed effect of different groups of sows, Fj = random effect of the farm, and ɛij = residual error. All comparisons were conducted using the R software, package lme4 (Boston, MA, RStudio Team: Integrated Development for R). A p < 0.05 was considered significant.

## Results

The incidence of SLS in the investigated sows was 47.8% (720/1507). Among five farms, the incidence of SLS varied between 44.3 % and 53.2%. The percentages of sows that reduced ≥5, 4, 3, 2, 1, and 0 piglets in the second parity were 7.8% (118/1057), 2.6% (39/1507), 5.9% (89/1507), 6.7% (101/1507), 11.8% (178/1057), and 12.9% (195/1507), respectively. The percentages of sows that increased 1, 2, 3, 4, and ≥5 piglets in the second parity were 11.1% (167/1507), 10.1% (152/1507), 7.8% (118/1507), 7.4% (111/1507), and 15.9% (239/1507), respectively.

Non-SLS sows had a 3.6 times higher risk of having DPBA3 than SLS sows (95% CI = 2.8–4.6; p < 0.001). Non-DPBA3 sows had a 2.7 times higher risk of having DPBA4 than DPBA3 sows (95% CI = 2.1–3.7; p < 0.001). Non-DPBA4 sows had a 3.2 times higher risk of having DPBA5 than DPBA4 sows (95% CI = 2.3–4.7; p < 0.001). However, compared with SLS sows, non-SLS sows had the same risk of DPBA4 (OR = 1.1, 95% CI = 0.8–1.5, p > 0.05) and DPBA5 (OR = 1.0, 95% CI = 0.7–1.5, p > 0.05). The overall incidence rates of DPBA3, DPBA4, and DPBA5 were 46.0% (178/577), 50.8% (398/783), and 56.5% (298/527), respectively. The percentages of sows that constantly increased PBA from parity 1 to parities 3, 4, and 5 were 19.0% (217/1140), 5.6% (44/783), and 0.6% (3/527), respectively. Meanwhile, the percentages of sows that constantly decreased PBA from parity 1 to parities 3, 4, and 5 were 15.6% (178/1140), 4.9% (38/783), and 1.1% (6/572), respectively.

SLS sows had a larger PBA1 than non-SLS sows (p < 0.001). However, PBA2 in SLS sows decreased to 10.9, whereas PBA2 in non-SLS sows increased to 14.4, resulting in a significant difference between these two groups (p < 0.001). From parity 3 to parity 5, PBA3, PBA4, and PBA5 did not differ between SLS and non-SLS sows (p > 0.05) (Table-1). On average, PBA2 was 0.8 piglets higher than PBA1 (12.7 vs. 11.9, p < 0.001); PBA3 was 0.7 piglets higher than PBA2 (13.4 vs. 12.7, p < 0.001); and PBA4 and PBA5 were 0.8 piglets higher than PBA3 (14.2 and 14.2 vs. 13.4, p < 0.001).

**Table-1:** Effect of SLS on numbers of piglets born alive, accumulative numbers of piglets born alive, and durations from farrowing 1 to different subsequent farrowings.

Parameters	Non-SLS sows	SLS sows	Effect size (95% CI)
PBA1 (piglets)	10.6 ± 2.9 (n = 787)^a^	13.3 ± 2.3 (n = 720)^b^	2.7 (2.3–2.9)
PBA2 (piglets)	14.4 ± 2.6 (n = 787)^a^	10.9 ± 3.1 (n = 720)^b^	−3.5 (−3.8–−3.2)
PBA3 (piglets)	13.5 ± 3.6 (n = 563)	13.4 ± 3.5 (n = 577)	−0.1 (−0.6–0.2)
PBA4 (piglets)	14.0 ± 3.7 (n = 378)	14.3 ± 3.5 (n = 405)	0.3 (−0.2–0.8)
PBA5 (piglets)	14.3 ± 3.5 (n = 259)	14.1 ± 3.3 (n = 268)	−0.2 (−0.7–0.4)
APBA1-2 (piglets)	25.0 ± 4.9 (n = 787)^a^	24.2 ± 4.7 (n = 720)^b^	−0.8 (−1.4–−0.4)
APBA1-3 (piglets)	38.3 ± 6.6 (n = 563)	37.7 ± 6.6 (n = 577)	−0.6 (−1.4–0.1)
APBA1-4 (piglets)	52.6 ± 8.7 (n = 378)	52.2 ± 8.5 (n = 405)	−0.4 (−1.7–0.8)
APBA1-5 (piglets)	67.1 ± 10.4 (n = 259)	66.7 ± 10.0 (n = 268)	−0.4 (−2.2–1.3)
APBA3-5 (piglets)	41.9 ± 7.1 (n = 259)	41.8 ± 7.0 (n = 268)	−0.1 (−1.3–1.1)
FI1-2 (days)	159.7 ± 35.0 (n = 741)	157.6 ± 30.2 (n = 673)	−2.1 (−5.7–1.1)
FI1-3 (days)	309.6 ± 42.7 (n = 557)	312.0 ± 44.5 (n = 507)	2.4 (−2.9–7.6)
FI1-4 (days)	462.8 ± 50.8 (n = 374)	464.4 ± 53.8 (n = 346)	1.6 (−6.1–9.2)
FI1-5 (days)	608.7 ± 48.3 (n = 249)	612.4 ± 58.6 (n = 233)	3.7 (−5.8–13.3)

PBA=Piglets born alive, APBA=Accumulative piglets born alive, FI=Farrowing interval, APBA1-n=Accumulative number of piglets born alive from parity 1 to parity n, FI1-n=Interval from farrowing 1 to farrowing n, SLS=Second litter syndrome, different superscripts in the same rows indicate the significant difference, ^a,b^Mean significance at p ≤ 0.001

Non-SLS sows had 0.8 piglets larger APBA1-2 than SLS sows (p < 0.001). However, when these sows farrowed in parities 3, 4, and 5, the numbers of APBA1-3, APBA1-4, and APBA1-5 became similar between the two groups (p > 0.05). The APBA3-5 in SLS and non-SLS sows was similar (41.8 vs. 41.9, p = 0.898). FI1-2, FI1-3, FI1-4, and FI1-5 did not differ between SLS and non-SLS sows (p < 0.05) (Table-1).

## Discussion

In later parities (3–5), PBA and APBA remained unaffected by SLS, whereas APBA1-2 was reduced. Until the fifth farrowing, there was no change in the FI by SLS.

About 47.8% of the investigated sows had an SLS incidence which was similar to reported values. The incidence of SLS at the sow level ranges from 33.3% to 55.8% [13, 15–19]. The high incidence of SLS in previous studies indicates that it is widespread in pigs of various breeds. Litter size in the second parity is negatively associated with absolute body reserves at first weaning and mobilization during lactation [26] and positively associated with body weight at first service [27]. Sows do not reach full corporal development during their first lactation and are susceptible to weight, fat, and protein loss [26]. High protein loss during lactation may impair ovarian function by decreasing the number of medium-sized follicles, reducing follicle fluid, and lowering the contents of estradiol and insulin-like growth factor-1 [28]. These impairments in ovarian function may result in decreased ovulation rates and/or increased embryonic loss, which subsequently causes SLS [16]. Better management, including proper feeding during gestation and prevention of excessive weight loss during lactation, is recommended to reduce SLS risk and increase farms’ profit [26].

The effects of SLS on PBA, APBA, and FI in this study agree with previous findings. Sell-Kubiak *et al*. [13] reported that SLS sows had 0.11–0.47 fewer piglets in later parities without significant difference and 1.34 fewer APBA1-2 piglets compared with non-SLS sows (p < 0.05). Saito *et al*. [18] found that APBA1-2 in SLS sows was reduced by 0.3 piglets and FI1-2 was reduced by 1.6 days compared with non-SLS sows. This pattern is similar to the finding in the present study, where APBA1-2 and FI1-2 in SLS sows were reduced by 0.8 piglets and 2.1 days (numerical difference in FI), respectively. These authors suggested that the reduction in the FI was a mechanism that counterbalanced the decreased APBA1-2 in the SLS sows [18]. However, this hypothesis cannot explain the relationship between APBA and FI when sows farrowed in parities 3–5 in the present study because the FI in SLS sows became numerically longer than that in non-SLS sows. Interestingly, sows with PBA2 either decreased by ≤2 piglets or increased by ≥4 piglets had a smaller APBA1-2 and lifetime APBA than other sows [25]. In this study, SLS and non-SLS sows can be classified as medium PBA1 + low PBA2 (ML) or low PBA1 + medium PBA2 (LM), respectively, according to Hoving *et al*. [22] and Gruhot *et al*. [14]. According to Gruhot *et al*. [14], ML sows had PBA1=10–12 piglets and PBA2 <11 piglets, LM sows had PBA1 <10 piglets and PBA2=11–13 piglets, and according to Hoving *et al*. [22], ML sows had PBA1=11–12 piglets and PBA2 <11 piglets and LM sows had PBA1 <11 piglets and PBA2=11–13 piglets. Gruhot *et al*. [14] found that APBA1-3, APBA1-4, and APBA1-5 were 0.4–0.8 piglets different between ML and LM sows. When sows were kept until parity 10, the APBA1-10 in both groups (ML, LM) were the same (111.7 piglets) [14]. Similarly, Hoving *et al*. [22] demonstrated that APBA1-5 and APBA3-5 were 0.7 and 0.3 piglets different between ML and LM sows, respectively. These results are similar to those in the present study because the numerical difference in APBA1-3, APBA1-4, and APBA1-5 between SLS and non-SLS sows was 0.4–0.6 piglets, and the APBA3-5 values in SLS and non-SLS were very similar. Therefore, SLS should not be used as an indicator of future low reproductive performance or for culling of sows.

Despite investigations, SLS’s impact on DPBA3 risk in sows remains unclear, as no studies on the topic have been conducted yet; however, intriguingly, SLS appears to decrease the likelihood of DPBA3 in sows. It has been demonstrated that skipping a cycle and inseminating sows at second estrus post-weaning [29] and increasing feed intake during the 1^st^ month of gestation in primiparous sows [30] can increase PBA2. None of the aforementioned methods have been studied for their impact on the risk of SLS. The effectiveness of these methods in decreasing DPBA3 occurrence remains uncertain. This study shows that the presence of DPBA3 decreases the risk of developing DPBA4, and the occurrence of DPBA4 reduces the risk of having DPBA5. The negative association between SLS and DPBA3, between DPBA3 and DPBA4, and between DPBA4 and DPBA5 suggests that there is fluctuation in PBA throughout sows’ lifetime production. After producing an increased PBA at a certain parity, sows seem more likely to produce a DPBA in the following parity, and after producing a DPBA at a certain parity, sows seem more likely to produce an increased PBA in the following parity. These findings, on the one hand, suggest that careful management should be conducted not only in primiparous sows to reduce the risk of SLS but also in multiparous sows to reduce the incidence of DPBA in later parities. On the other hand, considering the very small percentages of sows that either increased or decreased their PBA across 3^rd^, 4^th^, and 5^th^ successive parities (19.0%, 5.6%, and 0.6% for increases, and 15.6%, 4.9%, and 1.1% for decreases – it is possible to hypothesize that alternating increases and decreases in PBA could be a physiological strategy. The answer to this hypothesis is yet to be determined. If this hypothesis is supported, it will be challenging to identify an approach that can increase PBA for all successive parity. Furthermore, a sow with a DPBA at a single parity should not be considered a low reproduction; by contrast, the judgment should only be passed under the consideration of a combined PBA in at least two successive parities [25].

## Conclusion

From the 3^rd^ to the 5^th^ parity, SLS had no impact on the PBA and APBA or the FI. Sows’ survival should not be evaluated based on SLS. The production life of a sow exhibits a variable PBA pattern. The long-term production life of sows, as governed by the alternate decrease/increase pattern in PBA, remains uncertain and warrants further investigation. Using the data from commercial farms, this study had some limitations such as the bias in genotype, environment, herd health management, boar effect, and infectious diseases. Despite such limitations, this study provides valuable information on the effect of SLS on subsequent reproductive performance including PBA, APBA, and FI, and the effect of DPBA in parity n on the risk of DPBA in parity n+1.

## Authors’ Contributions

NHN, TK, DTKL, NVT, NDT, NCT, BVD, BTAD, and PS: Conceived and designed the study. NHN: Collected data. NHN and PS: Analyzed data, interpreted the results, and drafted and revised the manuscript. All authors participated in the scientific discussion and read, reviewed, and approved the final manuscript.
